# Robustness under parameter and problem domain alterations of Bayesian optimization methods for chemical reactions

**DOI:** 10.1186/s13321-022-00641-4

**Published:** 2022-09-01

**Authors:** Rubaiyat Mohammad Khondaker, Stephen Gow, Samantha Kanza, Jeremy G Frey, Mahesan Niranjan

**Affiliations:** 1 Department of Mathematics, University of Cambridge, Cambridge, UK; 2 Department of Chemistry, University of Southampton, Southampton, UK; 3 Department of Electronics and Computer Science, University of Southampton, Southampton, UK

**Keywords:** Bayesian optimization, Reaction optimization

## Abstract

The related problems of chemical reaction optimization and reaction scope search concern the discovery of reaction pathways and conditions that provide the best percentage yield of a target product. The space of possible reaction pathways or conditions is too large to search in full, so identifying a globally optimal set of conditions must instead draw on mathematical methods to identify areas of the space that should be investigated. An intriguing contribution to this area of research is the recent development of the Experimental Design for Bayesian optimization (EDBO) optimizer [1]. Bayesian optimization works by building an approximation to the true function to be optimized based on a small set of simulations, and selecting the next point (or points) to be tested based on an acquisition function reflecting the value of different points within the input space. In this work, we evaluated the robustness of the EDBO optimizer under several changes to its specification. We investigated the effect on the performance of the optimizer of altering the acquisition function and batch size, applied the method to other existing reaction yield data sets, and considered its performance in the new problem domain of molecular power conversion efficiency in photovoltaic cells. Our results indicated that the EDBO optimizer broadly performs well under these changes; of particular note is the competitive performance of the computationally cheaper acquisition function Thompson Sampling when compared to the original Expected Improvement function, and some concerns around the method’s performance for “incomplete” input domains.

## Introduction

Optimization problems are common throughout chemistry, for example optimizing the yield of a chemical reaction. Once an initial reaction pathway is determined, chemists wish to find reaction conditions that provide the best percentage yield—for example by varying the temperature or pressure at which the reaction takes place—to minimise the amount of input material required to produce the desired product. A closely related problem is that of reaction scope search, considering not just the physical conditions affecting the reaction but also the chemical space of reactants and catalysts used in the reaction itself.

Since chemical reactions can take hours or days to complete, it is infeasible to search through the entire set of possible chemical and physical configurations. Instead, a small subset of the search space is tested. Two common ways to determine such a subset are Design of Experiments [2] and Generalised Subset Design [3]. These methods identify important factors in the reaction yield and their optimal settings, and can thus be used to guide future experiments towards potentially high-yield configurations, but are not in themselves a complete solution for optimization.

An alternative approach which offers potential benefits over these methods is Bayesian optimization, an iterative global optimization algorithm based on statistical methods to identify potentially optimal settings of the inputs to an unknown function. Bayesian optimization has found success in hyperparameter tuning for machine learning models [4], where researchers face a similar issue of long model evaluation times. As a result, it has been recently applied to problems in chemistry, including the development of the Experimental Design for Bayesian optimization (EDBO) optimizer [1].

Bayesian optimization works by building a statistical model to approximate the function being optimized based on its knowledge of the function’s behaviour at previously-seen input conditions. This is termed the surrogate model, and can be built using a variety of statistical or neural network approximations. The surrogate model is based initially on a small set of test runs, but each new observation improves the quality of the approximation. The EDBO algorithm constructs the surrogate model using Gaussian Process regression [5], a method of smooth estimation which returns a probability distribution for the true output at an unseen set of conditions and thus takes account of uncertainty in its estimates. The distribution of possible values for a set of inputs given the observations seen so far is termed the posterior predictive distribution, and its variance depends on the distance between the location of the prediction and the nearest point at which the true value of the function is known from a previous run.

Selection of the next point to be tested is done using an acquisition function reflecting the value of points in the input space. There are several possible choices of acquisition function, which typically take account of a combination of both the average predicted output of each point in the input space and the uncertainty of the prediction. Bayesian optimization can be extended by selecting several sets of conditions to test at once instead of selecting points one at a time, a process called batched Bayesian optimization.

In this work, we aimed to investigate the robustness of the EDBO method to changes to its environment and parameters. In particular, many of our investigations focused on reducing the computational cost of the EDBO algorithm. The original paper used Expected Improvement (EI) as an acquisition function, which is somewhat computationally intensive. We therefore decided to consider the computationally cheaper Thompson Sampling (TS) method as an alternative. We also considered changes to the batch size in batched Bayesian optimization; again, larger batch sizes correspond to reduced computational overheads.

One of the main sources of variation in Bayesian optimization is the set of initial experiments conducted. In principle, given a set of initial experiments, the method is entirely deterministic unless the acquisition function or parameters of the surrogate model are changed. A poor selection of initial reaction conditions can affect the information learned by the surrogate model, so it is important to determine if the effect of this selection significantly hinders the ability of the EDBO optimizer to find the optimal conditions. We investigated this by considering the performance of the optimizer given extremely low-yield reaction conditions as its starting set.

In addition, we wished to consider the transferability of the EDBO optimizer across data sets and problem domains. To do this, we extracted two further reaction yield data sets from the literature and applied the method to these. We also applied the method to a data set in an entirely different problem area: the Harvard Clean Energy Project data set [6] of theoretical power conversion efficiency for millions of molecules in photovoltaic cells. Each of these new data sets posed its own challenges to the Bayesian optimization algorithm.

It is important to note that the work described in this paper was conducted using computational methods and existing data sets only. No attempt was made to exceed the best known reaction yields by using Bayesian optimization in conjunction with physical laboratory experiments, the task which would ultimately prove the truest test of the usefulness of these methods. Nonetheless, by investigating the optimizer’s applicability to different tasks and robustness to parameter changes and randomness, and by considering ways in which its computational overheads can be reduced, we hoped to make its use in future practical research more streamlined and efficient.

## Methodology

Abstractly, we are given some black-box objective function *f*(*x*) that we wish to minimise, which is expensive (time- and/or resource-intensive) to evaluate. We wish to optimize the objective function while minimising the number of evaluations needed. To do this, after taking some initial observations, we build a statistical model of the data, in this case a Gaussian Process regression model.

To choose the next point for evaluation, we use an acquisition function. There are many ways to define such a function; one example is Expected Improvement (EI) [7]. Given the current best observed value $x_+$, and our objective function *f*(*x*), define the Improvement Utility as

$$\begin{aligned}&\mathcal {I}(\mathbf {x}) = f(\mathbf {x}) - x_+ \texttt { if } f(\mathbf {x}) \ge x_+ \\&\quad\qquad \qquad \qquad \,\,\, 0 \texttt { if } f(\mathbf {x}) < x_+ \end{aligned}$$

Then, given the mean and variance at the point $\mathbf {x}$, the Expected Improvement is simply the average value of the Improvement Utility, according to the probability distribution defined by $\mathbf {x}$. However, commonly the improvement utility is reduced by some empirical exploration parameter $\delta$, which has the effect of discouraging small incremental improvements in local maxima in favour of continuing to explore the search space. This design choice is used in the EDBO optimizer [1].

An alternative acquisition function is Thompson Sampling (TS) [8], which samples the posterior predictive distribution given by the surrogate model and takes the point with highest objective value. This is less certain to arrive at an optimal value than Expected Improvement, but has the advantage of being computationally cheaper.

The key benefit of the acquisition function is that it is much easier to evaluate than the objective function, and so is easier to optimize. In particular, since our work was focused on discrete Bayesian optimization over tables of data, acquisition functions $\texttt {acq}(\mathbf {x})$ were optimized simply by evaluating them over the entire finite domain, and finding $\texttt {argmax}_{\mathbf {x} \in X} \texttt {acq}(\mathbf {x})$.

Then, we evaluate the objective function at the point selected by the acquisition function, and update the surrogate model with the new data. This concludes a round of optimization. Finally, the process is iterated until a fixed experiment budget is reached, a sufficiently high objective value is found, or it seems unlikely that further optimization will provide useful improvement. This concludes a full run of the optimizer.

**Fig. 1:**
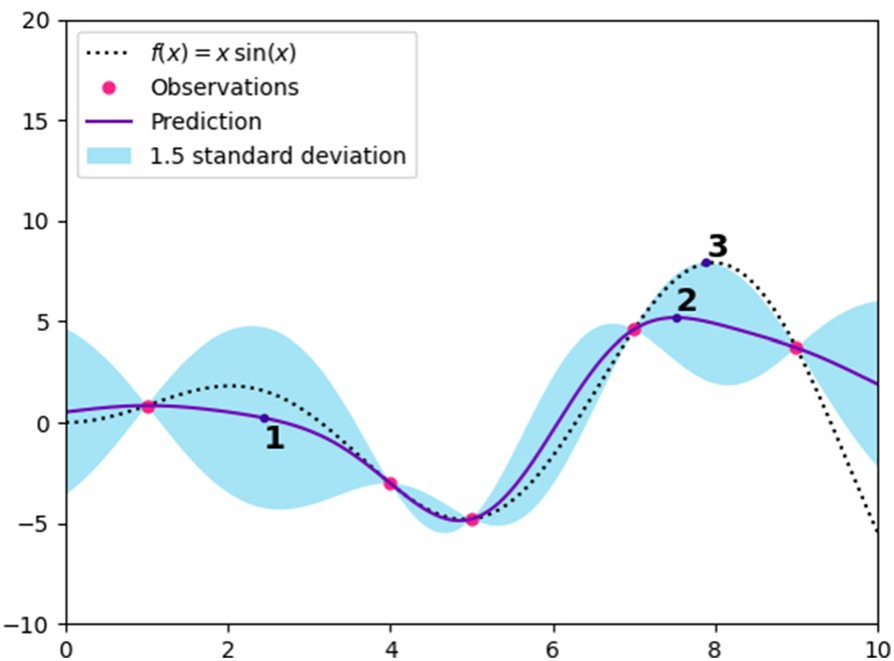
An example of a single round of Bayesian optimization for the function $f(x) = x \sin (x)$ with three different acquisition functions

Figure 1 provides an example of a round of optimization for the function $f(x) = x \sin (x)$. The surrogate model assigns each point in the domain a mean, and an uncertainty—note that the previous observations, represented by pink dots, have zero uncertainty. From this, several different acquisition functions could be used—maximal uncertainty (1), maximal predictive mean (2), or maximal predictive mean + uncertainty (3). Depending on the choice of function, one of these points will end up being the next observation. The model would then be recomputed at the end of the round.

### Batched Bayesian optimization

Conventionally, Bayesian optimization is done sequentially—the black-box function is evaluated at the chosen point, the model is immediately updated with the new data, and then the acquisition function is re-computed and maximised. However, chemists have the ability to run multiple reactions in parallel, with techniques such as High-Throughput Experimentation [9] allowing the evaluation of hundreds of different conditions at a time. Thus, in order to maximise the information gained by the model in a given amount of time, batched Bayesian optimization is carried out, where multiple points are chosen at each iteration to evaluate the objective function at.

Of course, this requires more guesswork on the part of the model at each iteration—the second evaluation point must be chosen without knowledge of the objective value at the first point. One common strategy, called Kriging Believer [10] is to estimate the value at the first point as the mean of the predictive distribution. Then, update the model with the estimate and determine the second point by re-computing and maximising the acquisition function. This is repeated until every desired point has been selected—the true values are then evaluated using the objective function, which replace the estimated values, and the round of optimization concludes.

One disadvantage of this method is that it is computationally sequential, since computing point $k+1$ requires estimates of the previous *k* points. While this is necessary for a deterministic acquisition function like EI, Thompson Sampling can be parallelized easily by taking multiple samples of the posterior predictive distribution, and so has a marked speed increase over EI for large batch sizes.

While this does reduce the computational cost, which is useful for the purposes of the project given that only simulations of the optimizer are run, in lab applications the limiting factor is likely to be the evaluation of the objective function itself (e.g. running the chemical reaction with the given conditions). The benefits of moving to TS with increased batch sizes in a real optimization environment are therefore likely to be limited.

### Optimization routine

A full run of the optimizer proceeds as follows.

1. A data set, acquisition function, batch size and experimental budget are provided.
2. The optimizer numerically encodes the search space.
3. The optimizer selects initial domain points to evaluate, by taking a random sample of the search space of size equal to the batch size. A seed can be supplied here to ensure reproducibility.
4. The optimizer conducts rounds of optimization until the experimental budget is reached.
5. The top *n* values obtained are output, where *n* was normally either 1 or 5.

In each case, the optimizer was tested by doing 50 full runs with different starting seeds, to gauge performance across a range of initial domain points.

### Numerical encoding

Before starting the optimization algorithm, the search space must be numerically encoded. For the problems considered in our work, this consists of computing molecular descriptors for chemicals given by SMILES strings. Three techniques were used for this—Density Functional Theory (DFT) data; Mordred [11], an open-source molecular descriptor calculator; and 512-bit Morgan Fingerprint Encoding [12], built-in to the rdkit Python module.

Once each chemical factor is encoded as a vector of descriptors, the encoding for the full configuration is obtained by concatenating all vectors. The overall vectors for each configuration are then listed as the rows of the matrix representing the full search space. Then, the EDBO optimizer pre-processes the data by removing columns that are highly correlated to save on memory.

## Results

### Suzuki–Miyaura and aryl amination data

The EDBO algorithm was originally developed [1] using data from two different reactions. The existing data sets on these two reactions are used in our work to investigate changes to the acquisition function and batch size parameters. The Suzuki–Miyaura reaction [13] is a cross-coupling reaction between a boronic acid such as indazole and an organohalide such as 6-bromoquinoline, with a Palladium catalyst. This reaction has important applications, being one of the most frequently used in pharmaceutical synthesis and wider medicinal chemistry [14]. Notably, Suzuki–Miyaura reactions have been the focus of recent work on optimization using machine learning methods, provoking both positive [15] and more skeptical [16] commentary. The data set studied consists of 5760 combinations of five variables: the pair of reactants, the ligand, the catalyst and the base present in the reaction. This is thus a problem of reaction scope search, as the variables which are altered to affect the reaction yield are fundamental to the chemistry of the reaction.

The second problem considered is the the Buchwald–Hartwig amination reaction [17], also with a Palladium catalyst. This is a cross-coupling reaction of amines and aryl halides for the synthesis of Carbon-Nitrogen bonds. It too has many important applications in the synthesis of a wide variety of compounds of importance to medicinal and materials chemistry [18]. This is again a reaction scope problem: 4608 conditions are available in the data set, consisting of different combinations of the aryl or heteroaryl halide, Buchwald ligand, base and isoxazole additive used in the reaction.

For the Suzuki–Miyaura and Buchwald–Hartwig aryl amination data sets, Density Functional Theory (DFT) data provided in the EDBO Github repository (https://github.com/b-shields/edbo) was used for numerical encoding of the search space, based on results from the paper that suggested this format minimised worst-case loss of the optimizer.

#### Batch size

The authors of the EDBO paper remarked that Expected Improvement with a batch size of 5 performed equally well to sequential Expected Improvement with the same experiment budget of 50. So, after verifying that the code provided from the EDBO paper functioned properly on the provided Suzuki and Aryl Amination data sets, we wished to determine to what extent the batch size used affected the optimizer performance, for a range of different sizes. In particular, since a larger size meant fewer rounds of optimization (with a similar total experimental budget) and more guesswork each round, we hypothesised that performance would degrade as batch size increased.

We opted to keep the experimental budget roughly constant, near 50. This entailed running the following experiments.

- Batch size 3: 17 rounds of optimization (budget 51).
- Batch size 4: 12 rounds of optimization (budget 48).
- Batch size 5: 10 rounds of optimization (budget 50).
- Batch size 6: 8 rounds of optimization (budget 48).
- Batch size 7: 7 rounds of optimization (budget 49).
- Batch size 8: 6 rounds of optimization (budget 48).
- Batch size 9: two separate experiments, with 5 and 6 rounds of optimization (budgets 45 and 54 respectively). In the results below, we present only the outcome of the runs with budget 54, since performance did not appear to differ significantly between the two sets of experiments.
- Batch size 10: 5 rounds of optimization (budget 50).

**Fig. 2:**
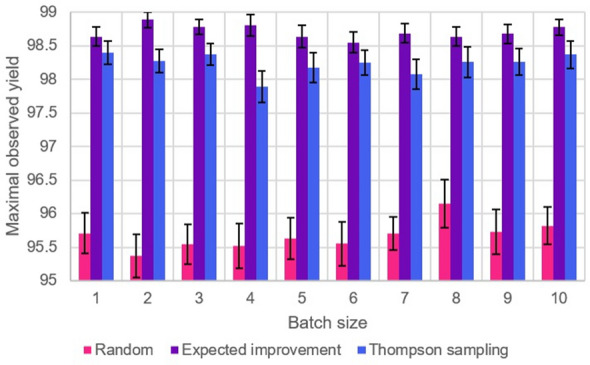
Graph of average optimizer performance (taken as the maximum observed yield after a full run, averaged over 50 runs), with standard error in the mean for the error bars, for the Suzuki reaction

We decided to test three different acquisition functions—EI, TS, and Random (which corresponded to ignoring the surrogate model and instead picking points at random), to serve as a control. Results for the Suzuki reaction are displayed graphically in Fig. 2. These indicate that there do not seem to be significant differences in performance across batch sizes. As an example, using Welch’s t-test to compare sample means with sequential EI, $p > 0.05$ for every other batch size when continuing to use EI. EI does consistently outperform TS, but importantly both methods significantly outperform the random control ($p < 0.05$ in all cases, using Welch’s t-test).

**Fig. 3:**
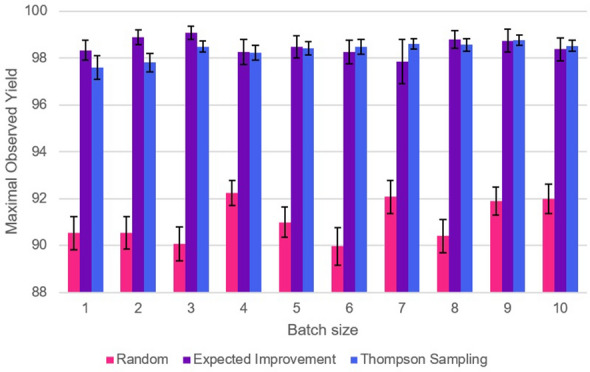
Graph of average optimizer performance (taken as the maximum observed yield after a full run, averaged over 50 runs), with standard error in the mean for the error bars, for the Aryl Amination reaction

Figure 3 displays the performance of the optimizer under different batch sizes and acquisition functions for the Aryl Amination reaction. As before, results indicate no significant dependence of performance on batch size, with both methods significantly outperforming the random control. Interestingly, Thompson Sampling occasionally outperforms Expected Improvement for certain sizes, which is likely an artefact of the search space of this particular reaction.

#### Altering set of initial experiments

**Fig. 4:**
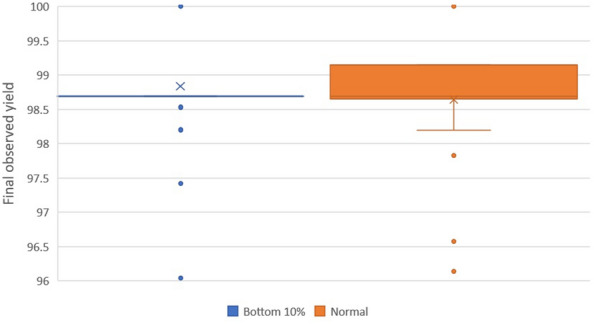
Box plots of optimizer performance for selecting from bottom 10% versus selecting normally for the Suzuki–Miyaura reaction with batch size 5, experimental budget 50, Expected Improvement acquisition function and 50 full runs conducted

After seeing that the optimizer performance was robust with respect to batch size, we moved on to testing to what extent the initial set of experiments given to the optimizer was important. The idea we explored was restricting this set to be chosen purely from the lowest 10% of experiments, ordered by reaction yield. These were taken for the Suzuki–Miyaura reaction, with a batch size of 5, and an experimental budget of 50, and an acquisition function of Expected Improvement, with 50 full runs conducted. These results are shown in Fig. 4. While average performance is very similar, it is interesting to note that the ‘bottom 10%’ method had identical lower and upper quartiles of $98.69\%$, suggesting the optimizer consistently found the same local maximum with this method.

### Iridium photocatalysts and palladium-catalysed cross-coupling reactions

**Table 1:** Performance of the optimizer with different acquisition functions on the Iridium photocatalysis data set

Acquisition Function	#Runs finding global maximum	#Runs finding second-highest
Random	11	19
Expected Improvement	23	27
Thompson Sampling	19	28

We further tested the optimizer on two unseen data sets, still within the domain of reaction yield optimization. Firstly, we looked at a paper investigating the rate constants associated with different Iridium photocatalysts when converting light into chemical energy for organic synthesis or chemical manufacturing [19]. The problem being investigated here is the relative catalytic activity of photocatalysts composed of iridium and different combinations of C⌃N and N⌃N ligands. The photocatalysts considered are built by combining one of 48 C⌃N ligands with one of 24 N⌃N ligands, and thus consists of 1152 combinations in total.

Next, we looked at a paper investigating a different variant of Palladium-catalysed Carbon-Nitrogen cross-coupling reactions, in this case ambient temperature reactions in a DMSO solvent [9]. Four inputs to the reaction scope are varied in this data set: the electrophile, nucleophile, catalyst and base. 6 electrophiles, 11 nucleophiles, 6 catalysts and 8 bases are considered in total, giving a complete search space of 3168 combinations of reactants and catalysts. However, the data set provided by the paper contains 1536 combinations: only 32 of the 66 possible combinations of electrophile and nucleophile were considered, each of which were then combined with all 48 combinations of catalyst and base. This poses a new challenge to the optimizer, as some configurations within the search space are “disallowed”.

For these two data sets, molecular structures of the relevant compounds were given visually in the paper without the associated SMILES—to obtain this, they were drawn using an online tool (http://www.cheminfo.org/flavor/malaria/Utilities/SMILES_generator___checker/index.html) that allowed output of the required SMILES. Once this was obtained, Mordred encoding was used to calculate molecular descriptors.

To test the optimizer on the Iridium photocatalyst data, we used an experiment budget of 50, with a batch size of 5, and again compare Expected Improvement, Thompson Sampling and the random control with 50 full runs of each. We report the results in Table 1. EI found the global maximum in $46\%$ of the runs conducted and the second-highest value in the remaining $54\%$. The performance of TS was slightly lower, at $38\%$ and $56\%$ respectively, but this nonetheless compares extremely well to the random acquisition function.

Investigations into the palladium-catalysed cross-coupling reactions data set were complicated by the absence of some of the possible reaction configurations not included in the data set. Initially, we chose to deal with this by giving the combinations that were ‘missing’ an area count of 0. Again, we used an experimental budget of 50, with a batch size of 5, comparing Expected Improvement, Thompson Sampling and the random control, with 50 full runs of each. This however led to fairly poor performance by the optimizer. This could have been due to missing combinations being labelled as 0 interfering with the model, especially if a missing combination was close to an optimal one in the search space.

**Fig. 5:**
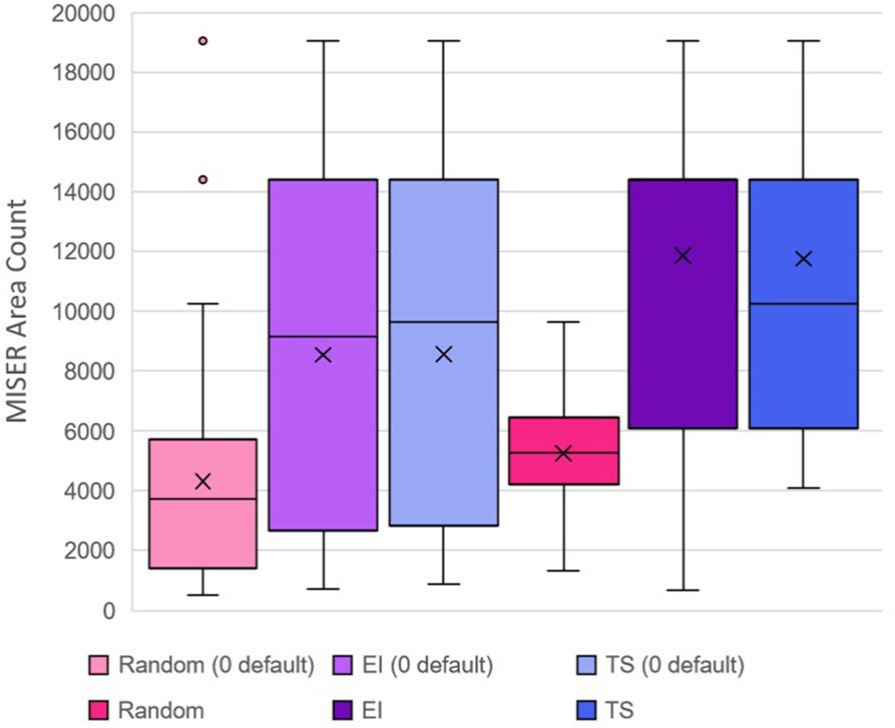
Box plots for the Palladium-catalysed cross-coupling data set. The “0 default” labels indicate those runs where missing combinations were coded as 0. Note that for regular Expected Improvement, the upper quartile of the data set was equal to the median, so an additional median line was not drawn

After this, the search space was modified to include only the 1536 combinations present in the data set, by manually providing the allowable configurations. The results of both approaches are shown in Fig. 5. As indicated by the figure, the revised approach led to markedly improved performance, which suggested that EDBO handled ‘missing values’ poorly overall and needed to be told the allowed domain points in advance.

### Harvard Clean Energy Project

Finally, after thoroughly testing the optimizer on multiple reaction yield data sets, we decided to see how it would fare on a very different kind of optimization problem. The Harvard Clean Energy Project (CEP) [6] was a computational screening of over two million molecules, with quantum chemistry calculations, in order to determine their theoretical power conversion efficiency (PCE) values for use in organic photovoltaics. This was both qualitatively and quantitatively different to what had been studied before, in that we were attempting to optimize a physical property using a single degree of freedom (which molecule we were testing) as opposed to varying multiple parameters for reaction yield optimization. The data set also includes other physical properties such as the molecular mass, short-circuit current density and open-circuit voltage, but these were not used in our work as the EDBO method relies on larger sets of molecular descriptors extracted using the methods described below. The full data set was too large to be loaded on a single computer, so we instead took a specific random sample of the data set of size 10,000 to investigate throughout.

Numerical encoding of the CEP data set produced a unique challenge, because the search space was one-dimensional, with the only factor being the candidate organic photovoltaic chemical. This was in contrast to the multidimensional reaction yield data sets we had investigated previously, whose size came from multiplying together combinations of different factors and not from a single factor having thousands of possibilities. Therefore, Mordred encoding was prohibitively slow as a sample size of 10,000 required computing molecular descriptors for 10,000 molecules. This led us to use 512-bit Morgan Fingerprint encoding instead, which was less comprehensive in detailing properties of molecules but significantly faster.

#### Initial results

**Fig. 6:**
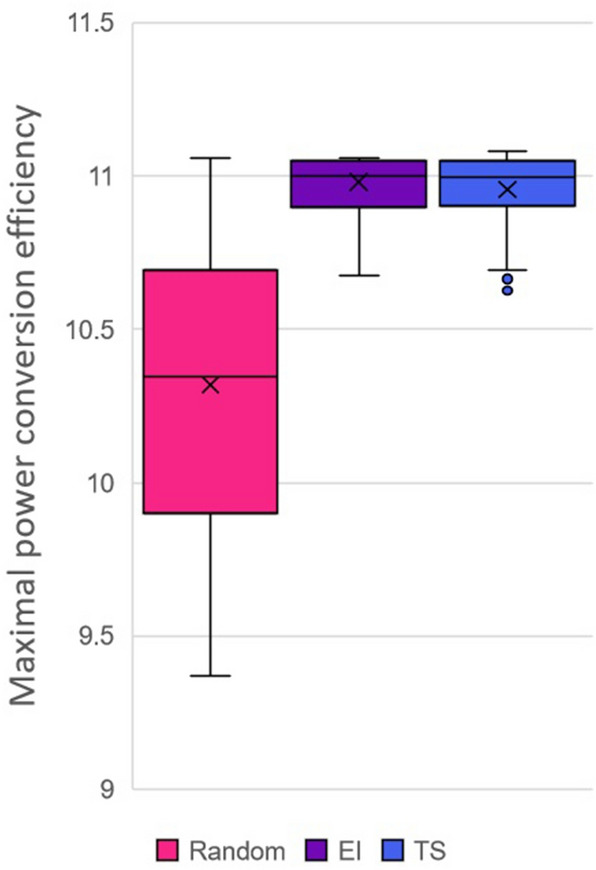
Performance of the optimizer with different acquisition functions on the Harvard PCE data set

In this case, we used an experimental budget of 100, with a batch size of 10, comparing Expected Improvement, Thompson Sampling and the random control, with 50 full runs of each. The results are displayed in Fig. 6. Expected Improvement and Thompson Sampling were remarkably similar, with no appreciable visual difference in the box plots of the results, and no significant difference ($p > 0.05$) when Welch’s t-test is used to compare their sample means. Both methods significantly outperformed random selection in terms of the mean PCE of the best molecule found by the optimizer ($p < 0.05$ using Welch’s t-test), which is encouraging given that this is an entirely different problem for the optimizer to tackle. In particular, the relative success of Thompson Sampling is worthy of note, as this method requires roughly one-quarter of the computational time of Expected Improvement for the CEP data.

**Fig. 7:**
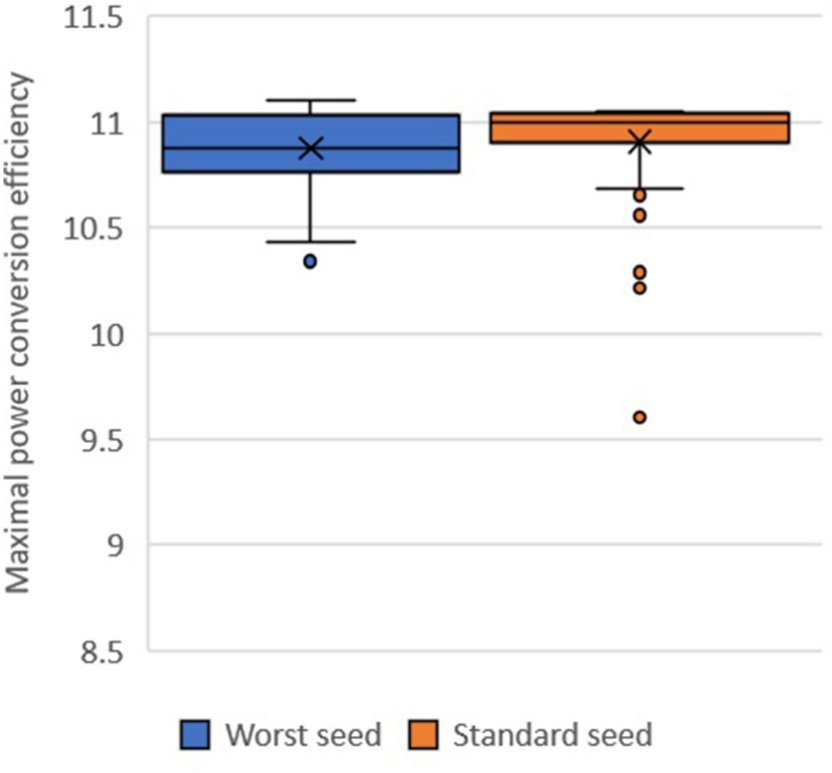
Performance of the optimizer with Expected Improvement acquisition function on the Harvard PCE data set for the worst seed (left) and median seed (right)

Since the optimizer performed so well on this initial subset, we wanted to see whether we could find a different subset of the same size where it performed more poorly. Subsets were determined by taking a random sample, which could be seeded, so we searched a few hundred seeds. To compare the subsets, 50 full runs of the optimizer were conducted, with a batch size of 10, experiment budget of 100, and with Expected Improvement as the acquisition function. Figure 7 shows the performance of the optimizer on the worst seed found during the search alongside the median seed. While optimizer performance is slightly poorer in the worst subset, the difference is relatively insignificant, indicating performance was fairly stable across different subsets.

#### Investigating different acquisition functions

After confirming that the EDBO optimizer could be applied successfully to this problem, we shifted to focusing on modifying the acquisition functions used in order to improve performance. In particular, we decided to look at improving the top 5 values returned by the optimizer, as opposed to merely the top value.

Our motivation for this was based on the calculated values being theoretical—therefore, it would be useful to have a large selection of molecules each of which had a good PCE as opposed to a single molecule with excellent PCE, to reduce the chances of relying heavily on an artefact of the PCE model.

The modification to the EI algorithm we explored was changing what value was being compared to for the sake of the Improvement Utility. Using the notation from earlier in the report, we altered the $x_+$ value used. Two modification strategies were evaluated:

- EI-*k*: Setting $x_+$ to be the *k*th highest value observed, with EI-1 representing ordinary Expected Improvement.
- E3I: Exploration Enhanced Expected Improvement [20]. In summary, it samples the surrogate model distribution multiple times, each time calculating Expected Improvement by setting $x_+$ to be the sample maximum, and then averaging the results. This tends to encourage more exploration of the sample space early on, and approaches normal Expected Improvement with more iterations.

**Fig. 8:**
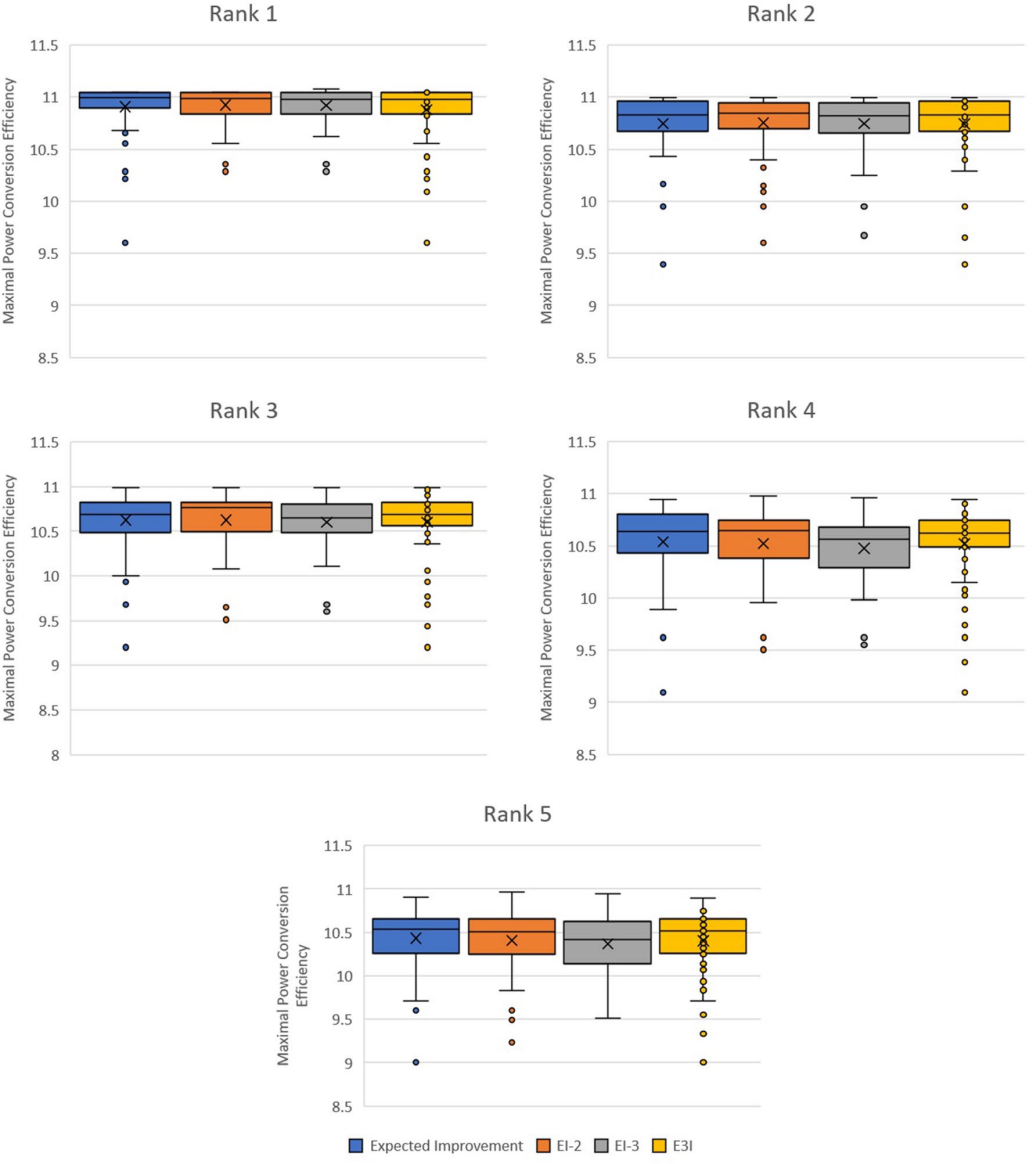
Distribution of obtained PCE for the top five molecules under four different acquisition functions in the Harvard data set

As is evident from the results in Fig. 8, each strategy performed fairly similarly, and no alternative strategy consistently performed better than standard Expected Improvement. There is however some evidence that the distribution of PCE for molecules at rank 3, 4 and 5 is more constrained when E3I is used, leading in particular to higher values in the third quartile. This may be a result of increased exploration of the sample space causing a range of high-performing molecules to be found more frequently.

## Conclusions and future work

The EDBO optimizer proved robust to a wide variety of changes made. Compared the original paper, performance was not severely impacted by either modifying the batch size, nor altering the initial experiments selected by the optimizer. Furthermore, the algorithm continued to perform well on subsequent reaction yield data sets, showing its applicability in its original problem domain. Moreover, the algorithm performed well when used for a different class of problem—optimizing a physical property of a single molecule—evidenced by its performance on the CEP data set. In general, this suggests both EDBO and similar optimizers designed for one chemical problem domain may find applicability in a large number of other domains, with minimal configuration, lessening the need for problem-specific algorithms.

Our results also demonstrate that a Thompson Sampling acquisition function consistently delivers a relatively similar level of performance to Expected Improvement. This is potentially important due to the speed advantages offered by Thompson Sampling through easy parallelization. For example, when investigating the Harvard data set with a batch size of 10, a round of TS took approximately one-quarter of the time to run as a round of EI. For very large data sets in which many rounds of optimization must be performed, it is therefore important to note that the faster Thompson Sampling method is competitive.

However, there are certainly limitations to the EDBO approach. In particular, as evidenced by the Palladium-catalysed cross-coupling data set, the algorithm handles ‘missing’ domain points poorly, and instead requires a fixed search space determined before optimization begins. Future work to allow the optimizer to instead adapt to both missing values and new candidate configurations may be useful. This may also have implications for potential use in physical experiments, where certain combinations of conditions may be infeasible to run in practice. In addition, efforts could be made to improve the acquisition function used to provide a selection of useful candidates, avoiding a sharp drop-off in quality across the top values.

Finally, it must be acknowledged that the experiments conducted in this research were exclusively computational in nature. It would be enlightening to test the optimizer in a physical lab setting to provide some hands-on data of its applicability to real-world reaction yield optimization, especially if yields surpassing the literature could be accomplished. This would also allow the optimizer to be tested at a larger scale, with hundreds of thousands of potential conditions, since the experiment budget itself would remain manageable, which might provide insights unobtainable from the data sets shown here. Using a more powerful computer, or computing cluster, to analyse a larger sample of the CEP data set would be a further test of the robustness of the algorithm in an unfamiliar problem domain, and could allow further room for experimentation on the acquisition function used.

## Data Availability

All code and data used in our work is available at https://github.com/Pseudonium/edbo.
